# Improving Actuator Wearing Using Noise Filtering

**DOI:** 10.3390/s22228910

**Published:** 2022-11-18

**Authors:** Paweł D. Domański

**Affiliations:** Institute of Control and Computation Engineering, Warsaw University of Technology, ul. Nowowiejska 15/19, 00-665 Warsaw, Poland; pawel.domanski@pw.edu.pl; Tel.: +48-22-234-7556

**Keywords:** noise filtering, loop performance, actuator weariness, valve travel, process control

## Abstract

Actuator, mostly valve, wearing is an important factor of the overall industrial control system operational cost. Actuator operational wear strongly depends on its operation. Highly utilized elements have a tendency to degrade faster. Therefore, the maintenance teams prefer to minimize their moves. In contrary, control engineers need the actuators to actively operate in their control loops to mitigate disturbances and follow the desired trajectories. Higher control performance is often achieved with an active use of actuators. Control loop quality depends on the controller setup and loop auxiliary functionality. Properly designed filtering not only facilitates controller action, but also impacts actuator operational wear. Industrial control templates are built using the blockware that is embedded in the existing control system. Distributed control system (DCS) and programmable logic controller (PLC) have a limited number of control algorithms. An engineer has to design the control structure and the associated sensor noise filtering using available functionality. This paper evaluates and measures the impact of noise filtering on the loop performance and on the actuator weariness. Relations between noise filtering time constant, loop performance and valve travel deliver recommendations for control engineers.

## 1. Introduction

The industrial univariate control loop template differs from the scientific textbook configuration. The raw single-element controller is often enriched with several auxiliary blocks and functionalities that make its operation robust and reliable. The overall system performance is improved, which results in a wider operating regime scope. These auxiliary functionalities consist of measurement noise filtering and conditioning for process variable and measured disturbances, actuator characteristics linearization, setpoint shaping, tracking, gain scheduling, AUTO/MANUAL mode switching, control error shaping and others. The orchestration of these elements allows reliable and robust loop operation, and gives the plant operator comfort and safety.

This work focuses on the measurement noise filtering. In general, the filtering, which depends on the loop positioning, might be organized in two ways. Independent univariate control loops need filtering on the process variable (PV) measurement input, as they are the origin of noises. Cascaded control configuration introduces a new degree of freedom for the downstream loop, as the noise can be also introduced through the setpoint. In such a case, PV feedback filtering will not work, and the filtering must be introduced into the control error signal, i.e., at the input to the controller. Such permanent fluctuations are transferred through the controller to its output and affect an actuator and the process [1]. Ref. [2] shows that the measurement noise has a serious impact on the control loop performance. Permanent fluctuations added to the measured process variable are transported through the negative feedback to the controller input as shown by [3]. This feature appears in any feedback control configuration. Process variable measurement filtering is used to counteract this issue. PID loops, being a majority of industrial control templates, seriously require effective filtering [4]. As the filters are implemented inside of the feedback loop, their design affects not only the loop performance, but PID tuning as well. Filtering should be implemented before the PID design.

One has to remember that any industrial control system, DCS or PLC, exhibits functional limitations. An engineer does not have unconstrained freedom in the selection of wished algorithms or approaches. A system-dependent embedded blockware list is limited to some basic (and popular) algorithms. This property also applies to the measurement noise filtering. Advanced filter strategies are often used in process industry, not only for measurement filtering, but also for data validation and reconciliation [5]. They are hardly available and cannot be used in many cases, though they would improve the process performance significantly. Moreover, they require specific expert knowledge, which is seldom available in industrial life. Control engineer may only use existing blocks, such as delay, lag, function generator, deadband (deadzone), hysteresis or saturation.

Filtering uses first-order lag and the function generator in most cases. This analysis focuses on the filter selection and its impact on the loop performance with a specific focus on the behavior of an actuator. The valve is the most popular actuator in the process industry. Once we consider its maintenance, we should evaluate some indicators that would measure its quality. One of the valve main weariness indicators is the measure, how much the valve is used, and how many position changes it encounters. The total path covered by the actuator, i.e., the valve in the considered case is called the valve travel, while the counter for its travel direction changes is called the valve stroke [6]. This measure is used as the main indicator for maintenance, allowing to detect element stiction or oscillations.

The rationale for this research comes from the industrial experience of, and discussions with, site control engineers. The subject has big practical importance, as expert tuners find it important to filter the feedback signal. The simulation analysis is selected intentionally, as the phenomenon is independent of the valve. It is even independent of the type of actuator. The same issue happens for dampers, pumps, ventilators, engines, etc. The research assumes the ideal actuator performance, which ideally realizes the controller output. The phenomenon appears in the feedback loop through the measurement noises, disturbances and cross-correlations between cooperating control loops in the multi-loop environment. The valve merely “lends” its name to the naming of the index. Nowadays, the subject starts to be even more important, especially if we bring the energy consumption into the picture. Lower “actuator travel” means lower energy consumption and smaller carbon footprint generated by the actuator. It should be taken into account as one discusses the energy-aware control system.

The aim of this paper is to analyze the effect of noise filtering on the overall loop performance (which should decrease with increasing the filter time constant) versus decreasing the valve travel index. Both configurations, i.e., PV and control error filtering, are investigated. Suggestions for filter design are given, showing how far an engineer can go with filtering without a heavy loss on a loop performance.

The paper starts with a description of the methods, i.e., filter design, control performance assessment (CPA) measures and valve travel index. The next section shows simulation results for PID benchmarks, while the last one concludes the work, showing possible open issues for further research.

## 2. Methods and Algorithm

The analysis uses methods and algorithms of engineering areas, such as filter design, CPA measures and valve travel index as a maintenance indicator.

### 2.1. Noise Filter Design

**Figure 1:**
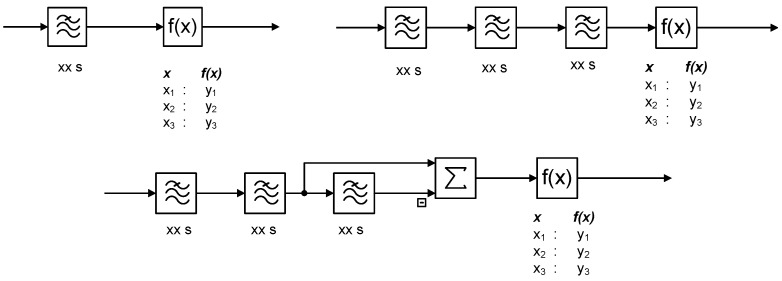
Noise filter industrial design configurations.

There are many approaches to filtering. The selected method depends on the engineering experience, procedures, good practices, control vendor templates or just the habits. Three exemplary industrial configurations for noise filtering used in process industry are sketched in Figure 1 using SAMA (Scientific Apparatus Makers Association) function blocks. They use first-order inertia (depicted as $\begin{array}{c} ≋ \end{array}$) and a static non-linear function generator denoted as $\begin{array}{c} f(x) \end{array}$  which allows to realize a static relationship parameterized by a set of *x-y* relation. The function generator realizes the deadband.

In the present research, the simplest $\begin{array}{c} ≋ \end{array}$ block configuration of a first-order filter (1) without the deadband is analyzed. It has a single tuning parameter: filter time constant $T_{F}$,

(1) $$G_{F}\left(s\right)=\frac{1}{T_{F}\cdot s+1}.$$

### 2.2. Loop CPA Measures

There are dozens of methods used in industrial CPA as shown in [7,8,9]. This work uses three indexes, which are frequently used as loop key performance indicators (KPI): mean square error (MSE), integral absolute error (IAE) and robust standard deviation $\sigma_{H}$.

Mean square error is the mean sum of the *N* points collected with some sampling period (time decrement) (2), while the integral absolute error is the mean sum of the absolute errors as in (3):

(2) $$MSE=\frac{1}{N}\sum_{k=1}^{k=N}\epsilon {\left(k\right)}^{2}=\frac{1}{N}\sum_{k=1}^{k=N}{\left[y_{0}\left(k\right)-y\left(k\right)\right]}^{2}$$

(3) $$IAE=\frac{1}{N}\sum_{k=1}^{k=N}\left|\epsilon (k)\right|=\frac{1}{N}\sum_{k=1}^{k=N}\left|y_{0}\left(k\right)-y\left(k\right)\right|$$

The ϵ(k) denotes the control error, $y_{0}\left(k\right)$ the setpoint and y(k) the process output.

#### Robust Statistics

If data are free of outliers, we may incorporate classical methods based on normal Gaussian distribution. In the opposite case, robust statistics, which aims at outliers removal, should be applied. It was introduced long ago, but [10] made it popular. A robust approach delivers location, scale and regression parameters estimates for time series infected with outliers. The M-estimator with logistic ψ-function is used.

### 2.3. Valve Travel Index

The valve travel index is a quantitative representation of how the valve moves in time. The valve travel index ($K_{VT}$) is calculated as a cumulative sum of absolute moves made by an actuator. This is a practical performance measure for a control loop, formulating one of the main measures for valve wear, giving indications of when the preventive maintenance activities should be performed [11]. Additionally, travel valve analysis is used to evaluate another indicator in the form of a number of direction changes in control valve travel per some time period ($K_{VS}$).

## 3. Simulations

**Figure 2:**
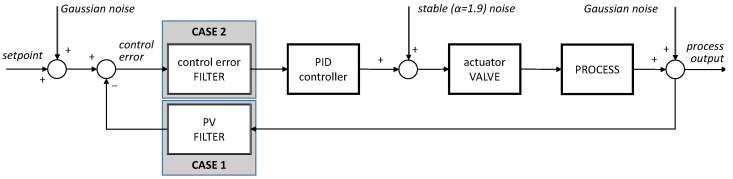
Simulation environment for noise filter analysis. The actuator device, the valve in the considered case, is separately isolated.

The analysis uses simulations carried out with a template shown in Figure 2. The PID control algorithm in the standard parallel form is applied as a benchmark algorithm. The standard octave function (optiPID.m) is used to find their optimal tuning. It uses an optimization routine that minimizes the weighted performance index, which incorporates three elements: the ITAE (integral time absolute error) criterion (weighting factor $\mu_{1}=1$), maximum overshoot ($\mu_{2}=10$) and sensitivity ($\mu_{3}=20$) [12]. The prepared template allows to investigate the effect of PV filtering in the univariate PID loop (CASE 1) and control error tuning in a downstream cascaded loop configuration. Each simulation consists of 1000 s sampled at 0.1 s.

There are several reasons behind such a choice. The main one says that the PID constitutes the majority (95 %) of industrial controls in process industry, as shown in [12]. Four PID benchmarks proposed by [13] are used:

- Multiple equal poles transfer function
  (4) $$G_{1}\left(s\right)=\frac{1}{{\left(s+1\right)}^{4}},$$
- A time-delay and double lag plant
  (5) $$G_{2}\left(s\right)=\frac{1}{{\left(0.2s+1\right)}^{2}}e^{-s},$$
- An oscillatory transfer function
  (6) $$G_{3}\left(s\right)=\frac{1}{\left(s+1\right)\left(0.04s^{2}+0.04s+1\right)}$$
- A non-minimum-phase process
  (7) $$G_{4}\left(s\right)=\frac{\left(1-\alpha \cdot s\right)}{{\left(s+1\right)}^{3}},\;\alpha =0.5.$$

Optimal controller transfer functions denoted by $R_{1}^{opt}\left(s\right)$ are designed. Table 1 shows the obtained tuning parameters.

### 3.1. Process Variable Filtering—Case 1

Process variable filtering analysis starts with multiple equal poles transfer function $G_{1}\left(s\right)$. Undisturbed and noisy scenarios are compared. Selected CPA measures are calculated and shown in Table 2 that compares ideal undisturbed loop with a noisy unfiltered one.

**Figure 3:**
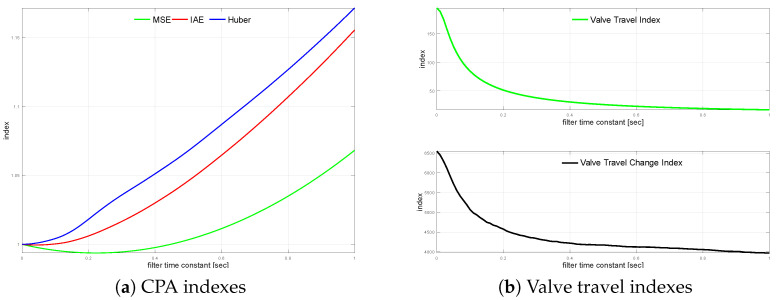
Process variable filtering relationships for $G_{1}\left(s\right)$.

MSE does not aim at the noises; the impact of measurement noise is small. It is due to the squared higher incidents, such as setpoint changes, which are much more important than the noise variations. Absolute error reflects the steady-state operation (and noises) more, while robust standard deviation represents noises at most. It is also visible that the noise has a tremendous effect on the actuator performance measured by indexes $K_{VT}$ and $K_{VS}$. Further, the controlled variable (CV) measurement filter is introduced. The loop is simulated using filter time constant values from $T_{F}=0$ s up to $T_{F}=1.0$ s incremented every 0.01 s. The filter impact on the loop is shown in Figure 3a with a relative index change versus unfiltered one. A similar relation for travel indexes is shown in Figure 3b. Even small values of the filter time constant improve the actuator performance, once, for larger values, the effect flattens.

MSE shows that the small time constant of the filter improves the loop performance, while slower ones ($T_{F}>0.45$) start to degrade the loop control quality. A similar effect is visible with IAE, but the range of loop improvement is more narrow and degradation starts sooner ($T_{F}>0.09$). Robust standard deviation starts to degrade just with the implementation of the filter. The effect of measurement filter on actuator performance is evaluated. Table 3 shows a comparison of the $T_{F}$ values: no filter, values when MSE and IAE start to degrade, and the value when $\sigma_{H}$ is degraded by 2.5% for $T_{F}=0.21$.

Undisturbed and noisy scenarios for a process with a time delay and double lag $G_{2}\left(s\right)$ are compared. Time trends are qualitatively similar for all plants and they are not further shown. CPA measures are evaluated and presented in Table 4. Similar to the previous $G_{1}\left(s\right)$ case, MSE does not aim at the noises, while IAE focuses on the steady-state noisy operation. As in previous case, the noise has a serious impact on actuator travel indexes $K_{VT}$ and $K_{VS}$.

**Figure 4:**
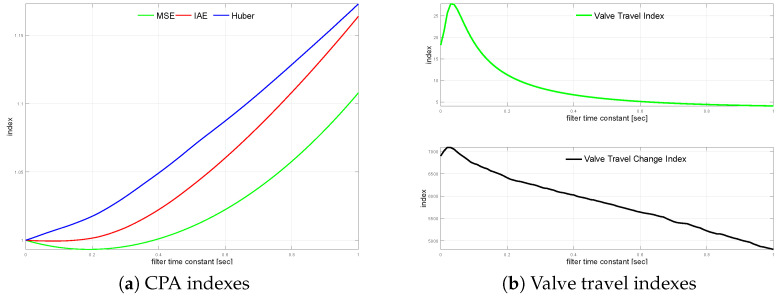
Process variable filtering relationships for $G_{2}\left(s\right)$.

The transfer function $G_{2}\left(s\right)$ loop is simulated using varying noise filter time constant values starting from $T_{F}=0$ s up to $T_{F}=1.0$ s incremented every 0.01 s. The measurement noise filter relative CPA index changes versus the unfiltered case on loop performance are shown in Figure 4a. The relationship for valve travel indexes $K_{VT}$ and $K_{VS}$ is sketched in Figure 4b.

In contrary to the previous case, valve travel index improvement starts for relatively high filter time constants. Valve travel indexes first increase ($T_{F}\in \left(0.0,0.1\right)$) and start to diminish with higher filtering. Additionally, the effect on $K_{VT}$ saturates, while change index $K_{VS}$ decreases rather slowly and linearly. Although this effect is rather strange and differs from an engineering intuition, it might be explained by the coordination between process (5) and filter time constants. The same effect can be seen in the next example as well (6), confirming such an explanation. MSE index shows that filters with a small time constant may even improve loop performance, while slower ones ($T_{F}>0.39$) start to degrade the loop control quality. The effect is visible with IAE but the range of loop improvement is narrow, and degradation starts sooner ($T_{F}>0.15$). Robust standard deviation does not show that effect and starts to degrade just with the smallest time constant of the filter.

Table 5 shows four filters, i.e., no filter, the value when MSE starts to degrade, the value when IAE starts decrease and the value when $\sigma_{H}$ degrades by 2.5% for $T_{F}=0.23$.

**Figure 5:**
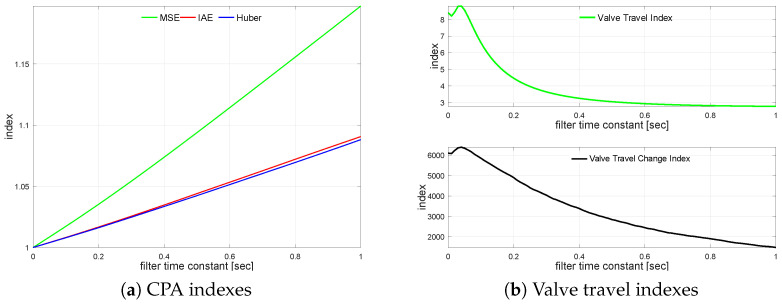
Process variable filtering relationships for $G_{3}\left(s\right)$.

Undisturbed and noisy scenario CPA indexes are presented in Table 6. They behave in the same way as for $G_{1}\left(s\right)$ and $G_{2}\left(s\right)$. The impact of the measurement noise filter on the loop performance is observed in the same way as for previous loops and is presented in Figure 5a,b. The results exhibit properties similar to $G_{2}\left(s\right)$. The explanation seems to be the same, as the process and filter are similar.

MSE starts to increase with a large value of the filter time constant $T_{F}=0.73$, while the same effect for IAE starts very soon with a small time constant $T_{F}=0.06$. The filter impact on actuator performance is shown in Table 7. Time constant $T_{F}=0.17$ reflects robust $\sigma_{H}$ degraded by 2.5%.

**Figure 6:**
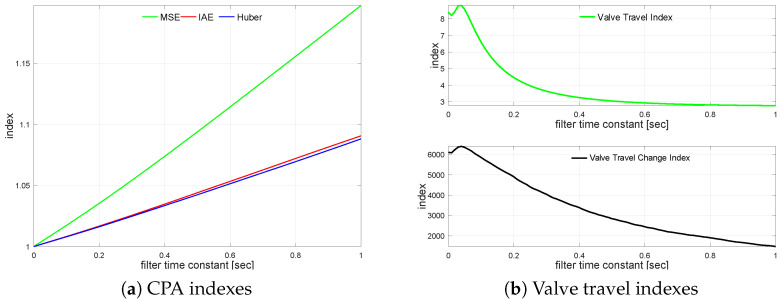
Process variable filtering relationships for $G_{4}\left(s\right)$.

A non-minimum-phase plant $G_{4}\left(s\right)$ behaves similarly to $G_{1}\left(s\right)$. The relationship between CPA measures and the measurement noise is presented in Table 8. The observed behavior is very similar to the previously analyzed plants. The measurement noise filter impact on loop performance is presented in Figure 6a. It shows the relative index change versus the unfiltered case. The relation for travel indexes is sketched in Figure 6b.

All indexes exhibit a constant increase almost linearly as the filter time constant increases. The measurement filter impact on actuator performance is shown in Table 9. Filter $T_{F}=0.18$ reflects $\sigma_{H}$ degraded by 2.3%. Shapes for both indexes exhibit a similar behavior with short initial rise followed by the exponential decrease saturating for slower filters.

### 3.2. Control Error Filtering

Control error filtering analysis starts with multiple equal poles transfer function $G_{1}\left(s\right)$. At first, scenarios of undisturbed and noisy environment are compared. CPA measures are calculated and presented in Table 10 that compare ideal undisturbed loop with the noisy unfiltered one.

Indexes show that MSE is not aiming at noises, as the impact of measurement noise is relatively small. It is due to the fact that because of the squared values higher incidents, such as setpoint, changes are much more important. Absolute error reflects the steady-state operation (and noises) more, while robust standard deviation reflects mostly noises. It is shown that the noise has a tremendous effect on the actuator performance measured by valve travel indexes $K_{VT}$ and $K_{VS}$.

**Figure 7:**
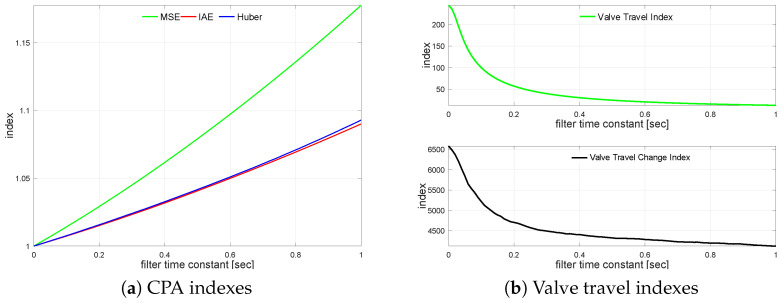
Control error filtering relationships for $G_{1}\left(s\right)$.

Next, the controlled variable measurement, i.e., the process output filter, is turned on. The loop is simulated with different filter time constants varying from $T_{F}=0$ s up to $T_{F}=1.0$ s incremented every 0.01 s. The impact of the control error filter on the loop performance is presented in Figure 7a. It shows relative index change versus the unfiltered case. A similar relationship for valve travel indexes is sketched in Figure 7b.

MSE features much faster degradation, compared to the other two indexes. Respective curves are close to the linear. In contrast, even filtering with a small time constant significantly improves the actuator performance, once, for larger time constants, the effect starts to flatten. The curve character is exponential. Thus adding of a small time constant should give much larger improvements in the valve operational wear, without serious loop performance degradation. This observation confirms the common practice that the filtering with a small time constant is always good for a loop. Finally, the effect of the measurement filter on the actuator performance is evaluated. Table 11 shows a comparison of the four filter $T_{F}$ values, i.e., no filter and values of MSE, IAE and $\sigma_{H}$ when they degrade by 2.0%. We see that robust estimator allows the largest time constants, as it does not focus on squared errors (similar to IAE). Slight 2.0% degradation in the loop performance gives tremendous improvement in valve travel (70∼80%) and a significant one in valve strokes (25∼30%).

Undisturbed and noisy CPA indicators are calculated and sketched in Table 12. Time trends are qualitatively similar for all plants, and they are not further shown. Similar to the previous $G_{1}\left(s\right)$ case, MSE is not aiming at the noises, while IAE focuses at the steady-state noisy operation. As in the previous case, the noise has a significant impact on both actuator valve travel indexes $K_{VT}$ and $K_{VS}$.

**Figure 8:**
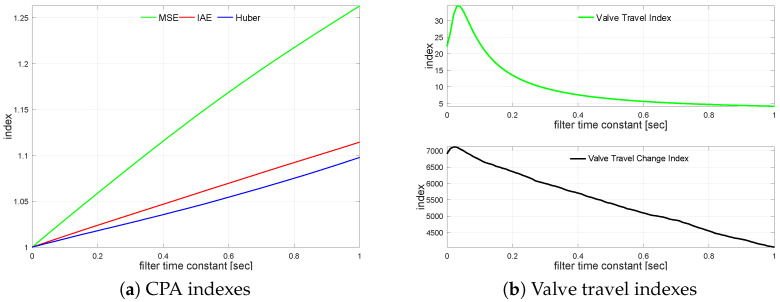
Control error filtering relationships for $G_{2}\left(s\right)$.

The transfer function $G_{2}\left(s\right)$ loop is simulated using varying control error filter time constant values from $T_{F}=0$ s up to $T_{F}=1.0$ s incremented every 0.01 s. The filter relative CPA index changes versus unfiltered case on loop performance are shown in Figure 8a. The relations for $K_{VT}$ and $K_{VS}$ are shown in Figure 8b.

In contrast to the previous case, valve travel indicator improvement starts for higher filter values. Operational wear indexes first increase ($T_{F}\in \left(0.0,0.1\right)$) and start to diminish with higher filter time constants. The effect on $K_{VT}$ saturates, while change index $K_{VS}$ decreases slower. Though this effect is rather strange and differs from an engineering intuition, it might be explained by the dynamic coordination between process (5) and filter time constants. A similar effect is seen in the next example.

All CPA indexes increase almost linearly. MSE has the highest increase gain, while the Huber robust index has the slowest one. It is due to the outliers and squared errors that are cut off by the robust M-estimator. The effect of the measurement filter on actuator performance is evaluated. Table 13 shows a comparison of four filter $T_{F}$ values, i.e., no filter, and the value when MSE, IAE and $\sigma_{H}$ degrade by 2.0%. We see that the previous effect is observed to a smaller extent; however, still, filtering improves the valve operational wear indicators. One has to be cautious with MSE, as it is misleading—higher filtering is required as indicated by MSE.

**Figure 9:**
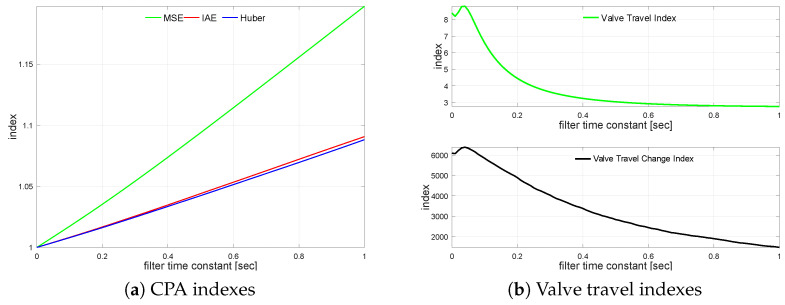
Control error filtering relationships for $G_{3}\left(s\right)$.

Undisturbed and noisy indexes in Table 14. The indexes behave in the same way as for $G_{2}\left(s\right)$ plant. The impact of the control error filter on the loop performance is evaluated similarly and is presented in Figure 9a,b. The results exhibit properties as in $G_{2}\left(s\right)$ plant. The explanation is the same, as the process dynamics and filter are quite close.

The filter effect on actuator performance is shown in Table 15. Highlighted values show degradation of the respective index by 2.0%. All CPA indexes increase almost linearly, with MSE exhibiting the highest gain and $\sigma_{H}$ the smallest.

Selected non-minimum-phase plant $G_{4}\left(s\right)$ behaves similarly to $G_{1}\left(s\right)$. The relation between CPA measures and the noise is shown in Table 16. The behavior is also very similar.

**Figure 10:**
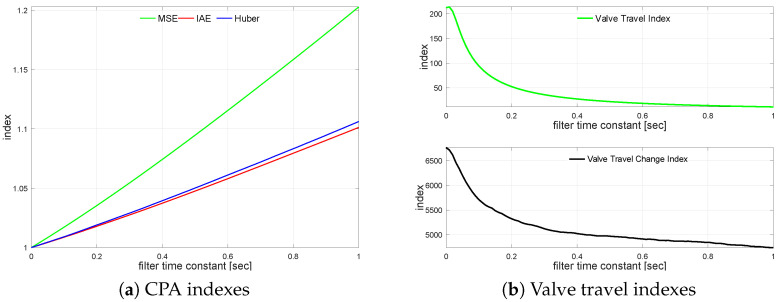
Control error filtering relationships for $G_{4}\left(s\right)$.

The impact of the measurement noise filter on the loop performance is presented in Figure 10a. It shows relative index change versus the unfiltered case. A similar relationship for valve travel indexes is sketched in Figure 10b.

As previously shown, all CPA indexes increase almost linearly, with MSE exhibiting the highest gain and $\sigma_{H}$ the smallest. The effect of the measurement filter on the actuator performance is presented in Table 17. Highlighted values show degradation of the respective index by 2.0%. The shapes for valve wear indexes show similar properties with exponential decrease and impact saturation for slower filters.

## 4. Conclusions and Further Research

The analysis addresses the impact of process variable measurement noise on the loop performance and the actuator wear. The noise causes permanent movement of the actuator. Industrial valve travel and valve stroke indexes $K_{VT}$ and $K_{VS}$ measure that effect. Simple but representative, first-order lag filtering is proposed as a solution. The results show a large impact of the process variable measurement noise on the loop performance and actuator travel.

Different CPA indexes may indicate unlike properties. Mean square error focuses on large deviations. Measurement noise effects are not well reflected by them. The MSE value first decreases with an introduction of the filter. Analogous impact on IAE exhibits with a smaller range, while robust standard deviation almost does not show such an effect. It can be explained with outlier robustness properties of the estimators. It is recommended not to use MSE in process variable noise analysis, as it is sensitive to setpoint or disturbance variations. A robust indicator, such as $\sigma_{H}$, is more suitable.

An impact of the process variable filter time constant on valve travel indexes reveals two other observations. Actuator travel indexes diminish with the increase in noise filter time constant and exhibit exponential decay. Thus, there is no need to use high filter time constant values, as they do not improve actuator weariness, while simultaneously degrading loop performance. The speed of decay differs between $K_{VT}$ and $K_{VS}$. An engineer decides which indicator is more appropriate.

There is an exception from that rule. In two cases, the general exponential decay is disturbed in a beginning, where travel indexes first increase for a short period of time. It appears for time-delayed and oscillatory processes. It may be explained by a existing correlation between the filter and the plant time constants. This observation raises some concerns and requires further cautiousness during the filter design. The noise filter design is not an easy compromise between the actuator wear benefits and the loop performance. The MSE might be misleading, and further research is required.

The control error filter design is also a compromise between the valve wear and loop performance. The difference is that CPA indexes increase monotonically, and small values of the filter time constant do not improve the loop performance. Proper loop design may improve actuator wear indicators, extending their operational period between maintenance activities.

## Figures and Tables

**Table 1:** Optimal PID settings for benchmarks.

	$k_{p}$	$T_{i}$	$T_{d}$
$R_{1}^{opt}\left(s\right)$	1.050	2.998	0.929
$R_{2}^{opt}\left(s\right)$	0.265	0.607	0.212
$R_{3}^{opt}\left(s\right)$	0.133	0.259	0.081
$R_{4}^{opt}\left(s\right)$	0.885	2.481	0.664

**Table 2:** $G_{1}\left(s\right)$ CPA indexes.

	Without Noise	Noisy	Relative Change
MSE	3.299	3.459	4.8%
IAE	0.500	0.647	29.4%
$\sigma_{H}$	0.261	0.479	83.5%
$K_{VT}$	8.3	195.3	2253%
$K_{VS}$	241	6546	2616%

**Table 3:** Filtering impact on $G_{1}\left(s\right)$ loop.

	$T_{F}=0.00$	$T_{F}=0.09$		$T_{F}=0.21$		$T_{F}=0.45$	
MSE	3.46	3.45	−0.4%	3.44	−0.6%	3.46	0.0%
IAE	0.65	0.65	0.0%	0.65	0.6%	0.67	3.7%
$\sigma_{H}$	0.48	0.48	0.2%	0.49	1.9%	0.59	5.9%
$K_{VT}$	195	91	−53%	50	−75%	28	−86%
$K_{VS}$	6546	5190	−21%	4526	−31%	4180	−36%

**Table 4:** $G_{2}\left(s\right)$ CPA indexes.

	Without Noise	Noisy	Relative Change
MSE	2.710	2.846	5.0%
IAE	0.505	0.624	23.6%
$\sigma_{H}$	0.370	0.545	47.3%
$K_{VT}$	2.6	18.2	600%
$K_{VS}$	1358	6900	408%

**Table 5:** Filtering impact on $G_{2}\left(s\right)$ loop.

	$T_{F}=0.00$	$T_{F}=0.15$		$T_{F}=0.23$		$T_{F}=0.39$	
MSE	2.85	2.83	−0.6%	2.83	−0.6%	2.85	0.0%
IAE	0.62	0.62	0.0%	0.63	0.3%	0.64	2.1%
$\sigma_{H}$	0.55	0.55	1.3%	0.56	2.0%	0.57	4.8%
$K_{VT}$	18	14	−23%	10	−45%	7	−63%
$K_{VS}$	6900	6566	−5%	6336	−8%	6036	−13%

**Table 6:** $G_{3}\left(s\right)$ CPA indexes.

	Without Noise	Noisy	Relative Change
MSE	2.129	2.316	8.8%
IAE	0.420	0.562	33.8%
$\sigma_{H}$	0.280	0.482	72.1%
$K_{VT}$	2.2	7.2	227%
$K_{VS}$	524	6156	1075%

**Table 7:** Filtering impact on $G_{3}\left(s\right)$ loop.

	$T_{F}=0.00$	$T_{F}=0.6$		$T_{F}=0.17$		$T_{F}=0.73$	
MSE	2.32	2.29	−0.9%	2.26	−2.3%	2.32	0.0%
IAE	0.56	0.56	0.0%	0.56	0.4%	0.62	9.6%
$\sigma_{H}$	0.48	0.29	−40.9%	0.49	2.1%	0.54	12.9%
$K_{VT}$	7	7	−1%	5	−38%	3	−60%
$K_{VS}$	6156	6282	2%	5526	−10%	2926	−52%

**Table 8:** $G_{4}\left(s\right)$ CPA indexes.

	Without Noise	Noisy	Relative Change
MSE	3.426	3.576	4.4%
IAE	0.482	0.630	30.7%
$\sigma_{H}$	0.263	0.477	81.4%
$K_{VT}$	6.8	172.7	2440%
$K_{VS}$	466	6870	1374%

**Table 9:** Filtering impact on $G_{4}\left(s\right)$ loop.

	$T_{F}=0.00$	$T_{F}=0.5$		$T_{F}=0.18$		$T_{F}=0.20$	
MSE	3.58	3.57	−0.1%	3.57	−0.1%	3.58	0.0%
IAE	0.63	0.63	0.0%	0.63	0.0%	0.64	0.8%
$\sigma_{H}$	0.48	0.48	0.4%	0.49	2.3%	0.49	2.5%
$K_{VT}$	173	123	−29%	50	−71%	46	−73%
$K_{VS}$	6870	6258	−9%	5302	−23%	5256	−23%

**Table 10:** $G_{1}\left(s\right)$ CPA indexes.

	Without Noise	Noisy	Relative Change
MSE	0.797	0.895	12.24%
IAE	0.711	0.742	4.40%
$\sigma_{H}$	0.898	0.926	3.12%
$K_{VT}$	186.5	244.5	31.1%
$K_{VS}$	6581	6583	0.0%

**Table 11:** Control error filter impact on $G_{1}\left(s\right)$.

	$T_{F}=0.00$	$T_{F}=0.14$		$T_{F}=0.26$		$T_{F}=0.27$	
MSE	0.90	0.91	2.0%	0.93	3.8%	0.93	4.0%
IAE	0.74	0.75	1.1%	0.76	2.0%	0.76	2.0%
$\sigma_{H}$	0.93	0.94	1.1%	0.94	2.0%	0.95	2.2%
$K_{VT}$	245	77	−68%	45	−81%	44	−82%
$K_{VS}$	6583	4933	−25%	4551	−31%	4529	−31%

**Table 12:** $G_{2}\left(s\right)$ CPA indexes.

	Without Noise	Noisy	Relative Change
MSE	0.935	1.028	9.94%
IAE	0.710	0.745	4.87%
$\sigma_{H}$	0.852	0.890	4.49%
$K_{VT}$	17.6	22.3	26.5%
$K_{VS}$	9614	6910	−0.1%

**Table 13:** Control error filter impact on $G_{2}\left(s\right)$.

	$T_{F}=0.00$	$T_{F}=0.7$		$T_{F}=0.17$		$T_{F}=0.23$	
MSE	1.03	1.05	2.0%	1.08	5.1%	1.10	6.8%
IAE	0.75	0.75	0.8%	0.76	2.0%	0.77	2.7%
$\sigma_{H}$	0.89	0.90	0.7%	0.90	1.6%	0.91	2.0%
$K_{VT}$	22	29	29%	16	−30%	12	−46%
$K_{VS}$	6910	6886	−0.3%	6464	−6.5%	6254	−9.5%

**Table 14:** $G_{3}\left(s\right)$ CPA indexes.

	Without Noise	Noisy	Relative Change
MSE	0.524	0.627	19.59%
IAE	0.564	0.611	8.24%
$\sigma_{H}$	0.694	0.746	7.47%
$K_{VT}$	6.7	8.4	26.1%
$K_{VS}$	5830	6104	4.7%

**Table 15:** Control error filter impact on $G_{3}\left(s\right)$.

	$T_{F}=0.00$	$T_{F}=0.12$		$T_{F}=0.24$		$T_{F}=0.25$	
MSE	0.63	0.64	2.0%	0.65	4.3%	0.66	4.5%
IAE	0.61	0.62	1.0%	0.62	2.0%	0.62	2.1%
$\sigma_{H}$	0.75	0.75	0.9%	0.76	1.9%	0.76	2.0%
$K_{VT}$	8	6	−29%	4	−52%	4	−52%
$K_{VS}$	6104	5648	−7%	4512	−26%	4402	−28%

**Table 16:** $G_{4}\left(s\right)$ CPA indexes.

	Without Noise	Noisy	Relative Change
MSE	0.815	0.904	10.8%
IAE	0.718	0.752	4.86%
$\sigma_{H}$	0.910	0.947	3.98%
$K_{VT}$	165.2	212.4	28.5%
$K_{VS}$	9819	6767	−0.8%

**Table 17:** Control error filter impact on $G_{4}\left(s\right)$.

	$T_{F}=0.00$	$T_{F}=0.12$		$T_{F}=0.21$		$T_{F}=0.23$	
MSE	0.90	0.92	2.0%	0.94	3.7%	0.94	4.1%
IAE	0.75	0.76	1.1%	0.77	2.0%	0.77	2.0%
$\sigma_{H}$	0.95	0.96	1.1%	0.97	2.0%	0.97	2.2%
$K_{VT}$	212	82	−61%	50	−76%	46	−78%
$K_{VS}$	6767	5600	−17%	5296	−22%	5246	−22%

## Data Availability

Not applicable.
